# Influence of vitamin D binding protein polymorphism, demographics and lifestyle factors on vitamin D status of healthy Malaysian pregnant women

**DOI:** 10.1186/s12884-020-03397-7

**Published:** 2020-11-23

**Authors:** Siew-Siew Lee, King-Hwa Ling, Maiza Tusimin, Raman Subramaniam, Kartini Farah Rahim, Su-Peng Loh

**Affiliations:** 1 Department of Nutrition, Faculty of Medicine and Health Sciences, Universiti Putra Malaysia, 43400 UPM Serdang, Selangor Malaysia; 2 Department of Biomedical Sciences, Faculty of Medicine and Health Sciences, Universiti Putra Malaysia, 43400 UPM Serdang, Selangor Malaysia; 3 Department of Genetics, Harvard Medical School, Boston, MA 02115 USA; 4 Prince Court Medical Centre, 50450 Kuala Lumpur, Malaysia; 5 Fetal Medicine and Gynaecology Centre (FMGC), 46200 Petaling Jaya, Selangor Malaysia; 6 Avisena Specialist Hospital, 40000 Shah Alam, Selangor Malaysia; 7 Research Centre of Excellence for Nutrition and Non-Communicable Diseases, Faculty of Medicine and Health Sciences, Universiti Putra Malaysia, 43300 UPM Serdang, Selangor Malaysia

**Keywords:** Vitamin D deficiency, Pregnant women, Single nucleotide polymorphism, Vitamin D binding protein

## Abstract

**Background:**

Vitamin D deficiency (VDD) has been related to vitamin D binding protein (*GC*) gene polymorphism, demographics and lifestyle factors in different populations. However, previous studies only focused on demographic and lifestyle factors or genetic factors alone. Therefore, this cross-sectional study aimed to assess the association between *GC* gene polymorphism, demographics and lifestyle factors with VDD among Malaysian pregnant women.

**Method:**

Information on demographic characteristics, dietary vitamin D intake from supplement and food, time spent outdoors, skin type and clothing were collected using a questionnaire. Plasma total 25-hydroxyvitamin D (25OHD) levels were measured using an Ultra-High-Performance Liquid Chromatography (UHPLC). Maternal *GC* single nucleotide polymorphisms (SNPs) (rs4588 and rs7041) were determined using restriction fragment length polymorphism (RFLP) technique.

**Results:**

Results showed that 50.2% of pregnant women were vitamin D deficient (25OHD < 30 nmol/L). VDD (25OHD < 30 nmol/L) was significantly associated with age, veiled clothing, maternal vitamin D intakes from both food and supplements, and *GC* rs7041(and *GC* diplotypes). In contrast to previous studies that reported for non-pregnant population, a significant positive association was found between CC genotype for SNP *GC* rs7041, *GC* 1s–1s and *GC* If-2 with risk of VDD (25OHD < 30 nmol/L).

**Conclusions:**

The high prevalence of maternal VDD found in this study suggests the need for urgent development and implementation of vitamin D supplementation or fortification strategies to reduce VDD among pregnant women. The discrepancy in the association between *GC* rs7041 gene polymorphism and VDD reflects the variation in the factors associated with VDD in pregnancy compared to non-pregnant state.

**Supplementary Information:**

The online version contains supplementary material available at 10.1186/s12884-020-03397-7.

## Background

Vitamin D is well known for its role in regulating bone metabolism by stimulating intestinal calcium and phosphorus absorption [1]. Recent findings showed that vitamin D also plays a vital role in cellular proliferation and regulation, trophoblast invasion, and immunomodulation at the maternal-fetal interface [2, 3]. Maternal vitamin D deficiency (VDD) has been suggested as a potential mechanism in mediating the pathogenesis of adverse pregnancy and neonatal outcomes, including preeclampsia [4, 5], gestational diabetes [6], small for gestational age [7, 8] and preterm birth [9].

Globally, it was estimated that 50–80% of pregnant women are deficient of vitamin D (25-hydroxyvitamin D (25OHD) < 50 nmol/L) [10]. Findings from previous epidemiological studies have demonstrated that VDD was high in countries that are located at a high latitude [10]. Recent studies also reported a high prevalence of VDD in countries with abundant sunshine such as Spain [11], India [12], Thailand [13, 14], and Malaysia [15]. In countries located at high latitudes such as the United Kingdom [16] and the Netherlands [17], a routine vitamin D supplementation is recommended for a specific group at risk, particularly pregnant women. Despite the high prevalence of VDD among pregnant women in the region with abundant sunshine like Malaysia, routine vitamin D supplementation has not been established due to insufficient data to inform vitamin D supplementation for pregnant women.

Previously, VDD has been related to environmental and lifestyle factors including latitude, season, sun exposure, skin type, clothing, dietary vitamin D intake, Body Mass Index (BMI) and ethnicity [18]. Several genome-wide association studies (GWAS) and candidate gene studies have shown that single nucleotide polymorphisms (SNPs) were associated with vitamin D status [19–22]. These SNPs located in or close to the genes encode the key enzymes or proteins for the metabolism, transportation and action of the mechanism of vitamin D, such as cytochrome P450-2R1 (*CYP2R1),* cytochrome P450-27B1 (*CYP27B1)*. Nonetheless, the studies related the association of the SNPs and vitamin D status were largely conducted for the non-pregnant women [19–22]. Also, previous studies focused on the factors associated with VDD during pregnancy were restricted to the study of only environmental [15, 23, 24] or genetic factors only [25–28].

In a review [29] that included a total of 120 genetic associations studies, it was reported that SNPs in *GC*, particularly rs7041 and rs4588 are the most reported SNPs associated with the level of 25OHD. Furthermore, SNP rs7041 and rs4588 are missense SNPs (functional SNPs) where the variation in the nucleotide resulted in the change in amino acid and vitamin D binding protein glycosylation pattern. For rs7041, A to C allele transversion causes the change of amino acid aspartic acid (CT**A**) to glutamic acid (CT**C**), whereas, for rs4588, G to T allele transversion causes the change of amino acid Threonine (T**G**C) to Lysine (T**T**C) [30]. The combination of the two SNPs (rs4588 and rs7041) has resulted in three different *GC* haplotypes/isoforms: *GC*1f (A allele rs7041 and G allele rs4588), *GC*1 s (C allele rs7041 and G allele rs4588) and *GC*2 (A allele rs7041 and T allele rs4588). These three *GC* haplotype/isoforms gave rise to 6 *GC* diplotypes: 1f/1f, 1s/1s, 2/2, 1f/1s, 1f/2, 1s/2 with different binding affinities and post-translational glycosylation [31, 32].

Besides being the major transport protein for all the vitamin D metabolites, vitamin D binding protein is important to keep 25OHD in the circulation pool [33]. Different *GC* isoforms have been linked to different vitamin D binding protein concentrations in postmenopausal women [33], infants and toddlers [34]. The variation in the level and functionality of vitamin D binding protein has been postulated to directly or indirectly affect the 25OHD level in the blood circulation. During pregnancy, the concentration of vitamin D binding protein is elevated by two to three folds compared to the non-pregnant (normal) state [35]. Thus, the effect of *GC* SNPs on vitamin D status among pregnant women may be different from that reported in the non-pregnant population. Therefore, this study aimed to (1) assess the prevalence of VDD, and (2) determine the associations between demographics, lifestyle and *GC* gene polymorphism (rs7041, rs4588 and the *GC* diplotypes) with VDD among healthy Malaysian pregnant women.

## Methods

### Study design and setting

This cross-sectional study was conducted from October 2015 through February 2017 at the Department of Gynaecology and Obstetrics Hospital Serdang, Selangor, Malaysia. Hospital Serdang is a public hospital located in Sepang district of Selangor, which caters for a population of about 2 million residents from three districts: Petaling, Sepang and Hulu Langat. This study adhered to the STROBE guidelines for observational studies.

### Participants

Pregnant women were conveniently recruited while they get admitted to the labour suite’s Patient Assessment Centre of Hospital Serdang for delivery. Inclusion criteria were Malaysian, aged 19 to 40 years, singleton pregnancy and week of pregnancy ≥37 weeks. Pregnant women who were diagnosed with pre-existing systemic disease, pregnancy complications, and had a history of bone and renal disorders were excluded from the study.

Using an estimated prevalence of vitamin D deficiency (< 25 nmol/L) in pregnant Malaysian women of 37% [23] and precision of 7% with a 95% confidence interval, the sample size was calculated to be 192 using the formula [36]: *n* = *Z*^2^*P*(1 − *P*)/*d*^2^. The addition of 20% for non-response gave a minimum sample size of 245 pregnant women.

### Data collection and blood sampling

Information on maternal ethnicity, education level, employment status and household income were self-reported using an interviewer-administered questionnaire. Maternal age, gestational age, last menstrual period (LMP), first booking (date, weeks, and ultrasound scan), gravidity, pre-pregnancy weight and heights were obtained from hospital electronic medical record and antenatal record. Gestational age was determined by LMP and confirmed by the first dating scan. Body Mass Index (BMI) was calculated as body weight divided by squared of body height (kg/m^2^). Venous blood was collected from pregnant women on the day of labour. All collected blood samples were processed within the collection day. After centrifugation, plasma was aliquoted, and buffy coat was collected for subsequent deoxyribonucleic acid (DNA) extraction.

The duration of exposure to sunlight per week was estimated using a questionnaire adapted from a previous study [37]. Pregnant women were asked about their outdoor activities from 7 am to 7 pm over the past 1 month. Information on the type of activity, duration (in minutes), usual outdoor attire, frequency (per week), use of glove, umbrella, and sunscreen were recorded. Based on the attire worn, the percent of body surface area (BSA) exposed to sunlight was estimated using the “Rule of Nine”.

In general, the climate in Selangor, Malaysia is warm to hot with an average daily sunshine of 6 h throughout the year. The region is typically affected by north-east monsoon (November–February; rainy season) and south-west monsoon (May to September, relatively dry season). Time spent outdoors and average available sunlight may vary between rainy and dry season [38]. As 25OHD has 3 weeks of half-life in blood circulation, exposure to sun for about 4 weeks before drawing the blood sample may better correlate with the circulating 25OHD. Therefore, in the present study, month of blood sampling was dichotomised into December to March and April to November.

To estimate the skin colour of the study participants, the Fitzpatrick scale [39] was used. Based on the women skin colour and skin tanning evaluation, study participants were classified into six different skin phototypes. In this study, as the sample size of each skin type subset was small, particularly skin type I, II, V and VI, skin colour types were dichotomised into light skin colour (Fitzpatrick scale I to III) and dark skin colour (Fitzpatrick scale IV and V) [40].

Daily vitamin D intake from dietary and supplemental sources was assessed using a vitamin D-specific semi-quantitative Food Frequency Questionnaire (FFQ) which was adapted from a previous study [41]. Pregnant women were asked to recall the brand (for commercial food), frequency and serving size of the listed food they had consumed over the past 1 month. At the same time, the pregnant women also provided the information regarding their supplemental intakes over the past 1 month: supplements (e.g. brand name, type of supplements, and specific nutrient), frequency and dosage of intake. To minimise the bias on depth and nature of probing, sun exposure and FFQ was administered by only one trained interviewer. The complete questionnaires used in this study is shown in Supplementary File 1.

### Measurement of plasma 25OHD

Plasma concentrations of 25OHD_3_ and 25OHD_2_ were determined using an ultra-high-performance liquid chromatography (UHPLC) and summed to calculate total plasma 25OHD. The inter-assay coefficient of variation at 50 nmol/L were 6 and 7% for 25OHD_3_ and 25OHD_2_, respectively. Multiple cut-offs (< 25, < 30 and < 50 nmol/L) were used to describe the prevalence of maternal VDD. The cut-off of National Academy of Medicine (formerly known as Institute of Medicine [IOM]) (< 30 nmol/L) which is associated with an increased risk of VDD [42] was used in the analysis of factors associated with maternal VDD.

### Genotyping

DNA was extracted from the buffy coat using the QIAamp DNA blood kit (QIAGEN, Germany) following to the manufacturer’s protocol. DNA yields and quality were determined using the NanoVue Plus UV spectrophotometer (GE Healthcare, USA).

Genotyping of *GC* SNPs rs4588 and rs7041 were carried out by restriction fragment length polymorphism (RFLP) technique. PCR was performed using a thermocycler (Thermo Fisher Scientific, USA). The PCR reaction mixture was prepared in a total volume of 20 μL: 10 μL of 2X GoTaq® G2 Green Master Mix (Promega, USA), 1 μL each of 10 nmol forward primer (5′-AAATAATGAGCAAATGAAAGAAGAC-3′) and reverse primer (5′- CAATAACAGCAAAGAAATGAGTAGA-3′), 3 μL of nuclease-free water and 5 μL of DNA (15 ng/μL). The PCR conditions for amplification included an initial step of denaturation at 95 °C for 10 min followed by 35 cycles of denaturation at 95 °C for 45 s, annealing at 51 °C for 45 s and elongation at 72 °C for 45 s, and finally a step of final extension step at 72 °C for 7 min. The PCR product, a 483-bp fragment was then digested separately using restriction enzyme, *HaeIII* (for rs7041) and *StyI* (for rs 4588) (New England Biolabs Inc., USA) in a total reaction volume of 20 μL. The digested products were then evaluated by electrophoresis on 1.5% agarose gel stained with ethidium bromide.

### Statistical analysis

Statistical analysis was performed using SPSS version 21.0 (SPSS Inc., Chicago, USA). All the variables have less than 5% missing data and single imputation technique was applied [43]. All the continuous variables were assessed for normality using skewness and kurtosis test. Mean and standard deviation (SD) were presented for normally distributed variables, whereas median and interquartile range (IQR) were presented for skewed variables. All the categorical variables were reported as numbers and proportions. Differences in the proportion of *GC* SNPs and diplotypes between ethnicity, and the Hardy-Weinberg Equilibrium (HWE) for each SNPs were examined using Chi-square test.

Individual associations between factors and VDD (25OHD < 30 nmol/L) were determined using univariate binary logistic regression. To further evaluate the joint associations of demographic, lifestyle and genetic factors with the risk of maternal VDD, multivariate logistic regression was performed. Variables that had *p*-value < 0.25 in univariate analyses and biologically important were included in multivariate analyses. Cramer’s V and Pearson’s correlation coefficient were used to test the associations between variable. Variables with r^2^ > 0.6 were considered highly correlated and excluded from the multivariate regression. Two separate multivariate models were constructed to determine the factors associated with VDD. Model 1 included selected demographic characteristics, lifestyle factors and the two *GC* SNPs (rs7041 and rs4588); Model 2 consisted of the demographics, lifestyle factors and *GC* diplotypes. All the statistical significance was specified at *p*-value < 0.05.

## Results

### Characteristics of participants

A total of 248 pregnant women provided informed consent to participate in the study. There were 217 of women had blood specimen, complete questionnaire and medical records, thus included in the analysis. Table 1 summarises the characteristics of study participants. The majority of study participants (86.2%) were Malays with the mean age of 29 ± 4 years. The percentage of participants with an education level up to tertiary education was slightly higher (56.7%) compared with secondary or lower (43.3%). Nearly 50% of the participants had a monthly household income in the range of RM3001 to RM5000 (approximately USD 750–1600). One-hundred and forty-one participants (65.0%) were enrolled between April and November (2015–2017), while 76 (35.0%) were enrolled in the study between December and March (2015–2017).

**Table 1:** Characteristics of study participants

Characteristics	*N* = 217
Ethnicity, n (%)	
Malay	187 (86.2)
Chinese	20 (9.2)
Indians and others^c^	10 (4.6)
Parity, n (%)	
Nulliparous	58 (26.7)
Multiparous	159 (73.3)
Maternal highest education level, n (%)	
Secondary and lower	94 (43.3)
Tertiary and higher	123 (56.7)
Household Income (per month), n (%)	
≤ RM3000	64 (30.0)
RM3001-RM5000	94 (44.1)
≥ RM5001	55 (25.9)
Month of sampling, n (%)	
December–March	76 (35.0)
April–November	141 (65.0)
Fitzpatrick skin type, n (%)	
Light (Type I, II, III)	97 (44.7)
Dark (Type IV, V &VI)	120 (55.3)
Veiled, n (%)	
Yes	179 (82.5)
No	38 (17.5)
Use of supplement containing vitamin D, n (%)	
Yes	87 (40.1)
No	130 (59.9)
Maternal age (in years) ^a^	29 ± 4
Week of pregnancy^a^	39.1 ± 1.1
Pre-pregnancy BMI (kg/m^2^) ^a^	23.7 ± 5.1
Time spent outdoors per week (hours)^b^	1.9 (0.7–4.1)
% BSA exposed to sunlight^b^	6.5 (6.5–6.5)
VD intake from food (μg/day) ^a^	8.3 ± 5.0
VD intake from supplement (μg/day) ^a^	3.8 ± 5.6
Plasma Total 25OHD (nmol/L) ^b^	29.8 (18.8–43.5)

Note: Categorical variables are presented as n (percentages); ^a^variable is presented as mean ± standard deviation; ^b^variables are presented as median (first quartile, third quartile)

^c^Others included Kadazan (*n* = 1), Bajau (*n* = 1), Suluk (*n* = 1) and mixed ethnic (*n* = 1)

The median time spent outdoors per week was 1.9 (range: 0.7–4.1) hour. As the majority of the study participants were Malay, about 82.5% of participants were veiled. Median % BSA exposed to sunlight was 6.5%, which was consistent with the usage of veiled clothing (covered on the head, with long sleeve and long pants) and sandals. Not more than half of the respondents (40.1%) consume vitamin D containing supplements.

Table 2 shows the distribution of the *GC* SNPs and diplotypes in the study participants. The genotype distribution for the two SNPs were in Hardy-Weinberg equilibrium (χ^2^ < 0.3841, *p* > 0.05). Minor allele frequencies for *GC* rs4588 (T allele) and *GC* rs7041 (C allele) were 21 and 37%, respectively. The results showed heterogeneity of the allele frequencies for the two *GC* SNPs between ethnicity. The frequency of the rs4588 T allele was significantly higher in Chinese (35.0%) than Malay, Indians and other ethnicities (19.0%) (χ^2^ = 4.904, *p* < 0.015). The *GC* rs7041 C allele showed the trend of lower frequency among Chinese (23.0%) than non-Chinese (38.0%) participants, although the difference was not statistically significant (*p* = 0.051). For *GC* diplotypes, 1f-1s was the most frequent diplotypes in pregnant women but diplotypes 2–2 was found in the lowest frequency (2.8%) in the study population.

**Table 2:** Distribution of *GC* genotypes and diplotypes by ethnicity

		Total (***n*** = 217)	Malay, Indians & others (***n*** = 197)	Chinese (***n*** = 20)	χ^**2**^	***p***-value
***GC*** **rs4588**						
Allele	G	(79.5)	(81.0)	(65.0)	4.909	0.015
	T	(20.5)	(19.0)	(35.0)		
Genotype	GG	135 (62.2)	127 (64.5)	8 (40.0)		
	GT	76 (35.0)	66 (33.5)	10 (50.0)		
	TT	6 (2.8)	4 (2.0)	2 (10.0)		
***GC*** **rs7041**						
Allele	A	(63.4)	(62.0)	(77.0)	3.793	0.051
	C	(36.6)	(38.0)	(23.0)		
Genotype	AA	89 (41.0)	78 (39.6)	11 (55.0)		
	AC	97 (44.7)	88 (44.7)	9 (45.0)		
	CC	31 (14.3)	31 (15.7)	0		
***GC*** **diplotypes (rs4588 + rs7041)**						
Allele	1f	(43.1)	(43.1)	(42.5)	6.934	0.031
	1 s	(36.6)	(38.1)	(22.5)		
	2	(20.3)	(18.8)	(35.0)		
Diplotypes	1f-1f	44 (20.3)	40 (20.3)	4 (20.0)		
	1s–1s	31 (14.3)	31 (15.7)	0 (0.0)		
	1f-1s	60 (27.6)	56 (28.4)	4 (20.0)		
	1f-2	39 (18.0)	34 (17.3)	5 (25.0)		
	1s-2	37 (17.1)	32 (16.2)	5 (25.0)		
	2–2	6 (2.8)	4 (2.0)	2 (10.0)		

Categorical variables are presented as n (percentages).

### Prevalence of VDD

The median (IQR) total 25OHD concentration was 29.8 nmol/L (Q_1_-Q_3_ = 18.8–43.5 nmol/L), with prevalence of vitamin D status among pregnant women as follows: < 25 nmol/L (41.9%), < 30 nmol/L (50.2%) and < 50 nmol/L (82.2%).

### Factors associated with maternal vitamin D deficiency

Logistic regression analyses showed that the risk factors for VDD (25OHD < 30 nmol/L) were higher maternal age and veiled clothing (*p* < 0.05) (Table 3). In contrast, vitamin D intake from food, supplements and the %BSA were the protective factors (*p* < 0.05).

**Table 3:** Associations between demographic and lifestyle factors with maternal VDD (25OHD < 30 nmol/L) in univariate analysis

Covariates	Crude OR	95% CI	***p***-value
Ethnicity			
Malay, Indians and others	2.56	0.94–6.93	0.065
Chinese	1.00	Reference	
Parity			
Nulliparous	0.90	0.49, 1.64	0.728
Multiparous	1.00	Reference	
Maternal highest education level			
Secondary and lower	0.98	0.58–1.68	0.953
Tertiary and higher	1.00	Reference	
Household Income (per month)			
≤ RM3000	0.72	0.35–1.49	0.373
RM3001-RM5000	0.63	0.32–1.24	0.181
≥ RM5001	1.00	Reference	
Month of sampling			
December–March	0.66	0.37–1.15	0.142
April–November	1.00	Reference	
Fitzpatrick skin type			
Light (Type I, II, III)	1.00	Reference	
Dark (Type IV, V &VI)	0.63	0.36–1.07	0.087
Veiled			
Yes	2.56	1.22–5.40	0.013
No	1.00	Reference	
Maternal age	1.08	1.01–1.16	0.018
Week of pregnancy	0.93	0.74–1.18	0.572
Pre-pregnancy BMI	1.02	0.97–1.08	0.441
Time spent outdoors per week (h)	0.68	0.35–1.33	0.262
BSA exposed to sunlight	0.32	0.11–0.95	0.039
VD intake from food	0.93	0.88–0.99	0.014
VD intake from Supplements	0.93	0.89–0.98	0.008

*BMI* Body mass index, *VD* Vitamin D

For genetic factors, the results revealed that the homozygous mutant (CC genotype) of *GC* rs7041 was associated with an increased risk of VDD when compared to other genotypes (Table 4). Nonetheless, no significant association was found between VDD and SNP *GC* rs4588. *GC* diplotype (combinations of rs7041 and rs4588) 1s–1s and 1f-2 were associated with an increased risk of VDD.

**Table 4:** Associations between *GC* genotypes and diplotypes with maternal VDD (25OHD < 30 nmol/L) in univariate analysis

		Crude OR	95% CI	*p*-value
*GC* rs4588	GG	1.00	Reference	
	GT + TT	1.07	0.61–1.87	0.814
*GC* rs7041	AA	0.65	0.37–1.17	0.654
	AC	1.00	Reference	
	CC	2.87	1.22–6.74	0.016
*GC* diplotype	1f-1f	1.00	Reference	–
	1s–1s	2.76	1.06–7.22	0.038
	1f-1s	1.07	0.49–2.36	0.854
	1s-2	0.80	0.33–1.96	0.626
	1f-2	2.40	0.97–5.70	0.059
	2–2	1.30	0.24–7.26	0.753

Variables that fulfilled the criteria (*p*-value < 0.25 in univariate analyses and biologically important) were maternal age, ethnicity, household income (RM3001-RM5000), month of sampling, vitamin D intake from food and supplements, Fitzpatrick skin type, percent BSA exposed to sunlight, veiled clothing, SNP rs7041, SNP rs4588 and *GC* diplotypes.

Strongly correlated variables were excluded to avoid the possibility of multicollinearity problem. As % BSA exposed to sun and veiled clothing were strongly correlated, a decision was made to include only veiled in the multivariate analysis. Given that ethnicity was overlapped with the variables veiled and skin colour, ethnicity was not included in the multivariate analyses. Based on the univariate analysis, *GC* rs7041 appeared to have a recessive effect on vitamin D status, while rs4588 have a dominant effect. Thus, the recessive and dominant models were accounted for SNP rs7041 and rs4588, respectively in the multivariate models. Therefore, the final multivariate models included: age, household income (RM3001-RM5000), the month of sampling, vitamin D intake from food, vitamin D intake from supplements, Fitzpatrick skin type, veiled, *GC* rs704 and rs4588 genotype (or diplotypes).

In the backward stepwise multivariate analysis (model 1; Table 5), maternal age, vitamin D intake from food, vitamin D intake from supplements, veiled, *GC* rs7041 SNP and month of blood sampling were significantly associated with VDD (Table 5). The results demonstrated that the risk of VDD increased as the age increased but decreased as the vitamin D intake from food and supplements increased. Pregnant women who were veiled had about 4 times higher risk of VDD compared to pregnant women who did not veil themselves. Likewise, pregnant women who carried homozygous mutant in *GC* rs7041 SNP had about 3 times higher risk to have VDD compared with other women who carried other genotypes. In a separate multivariate model (model 2), the analysis showed that pregnant women who had diplotypes 1f-2 and 1s–1s both had 3–4 times higher risk of VDD compared with pregnant women of other diplotypes. Time spent outdoor, skin type and other factors were not significantly associated with maternal vitamin D status.

**Table 5:** Multivariate models of demographic, lifestyle, *GC* genotypes and diplotypes with VDD (25OHD < 30 nmol/L)

Variables	Model 1			Model 2		
	aOR	95% CI	***p***-value	aOR	95% CI	***p***-value
Age	1.11	1.03, 1.19	0.006	1.11	1.03, 1.20	0.005
Dietary VD intake	0.89	0.84, 0.96	0.001	0.89	0.83, 0.95	< 0.001
Supplemental VD intake	0.92	0.87, 0.98	0.004	0.91	0.86, 0.97	0.003
Veiled (Yes)	4.27	1.80, 10.11	0.001	4.65	1.92, 11.25	0.001
Month of sampling (Dec-Mar)	0.50	0.26, 0.94	0.030	0.52	0.28, 0.99	0.047
*GC* rs7041	2.96	1.13, 7.76	0.028	–	–	–
*GC* rs4588	0.56	0.29, 1.08	0.084	–	–	–
*GC* Diplotype 1f-2	–	–	–	3.75	1.60, 8.77	0.002
*GC* Diplotye 1s–1s	–	–	–	3.08	1.19, 7.99	0.021

## Discussion

Malaysia is a tropical country where sunlight is available throughout the year. However, the prevalence of VDD among pregnant women is high. An estimate of 82.0% of maternal plasma 25OHD < 50 nmol/L was consistent with previous studies conducted in the local urban area (Kuala Lumpur), where 90 and 72% of VDD (25OHD < 50 nmol/L) were found among pregnant women in the first trimester [15] and at delivery [24], respectively. The VDD prevalence reported in the present study (82.2%) was higher compared to the previous study (61.0%) conducted among pregnant women in a private hospital in Kuala Lumpur [44]. The variation could be attributed to differences in the proportion of ethnicity and socio-economic background among study participants in present and previous studies. The prevalence of maternal VDD reported in the present study was comparable to that reported in countries located at high latitude, which included Ireland (80.0%) [45] , Germany (77.0%) [46] , China (75.0%) [47] and Japan (73.0%) [48]. Nevertheless, the VDD prevalence found in the present study was higher compared to that reported in Thailand (20–40%) [13, 14] and India (66.0%) [12] among pregnant women at delivery and third trimester.

In previous studies, the factors that had been reported to be significantly associated with maternal vitamin D status differ accordingly [49] owing to vitamin D status is country-, ethnic- or subgroup-specific. Besides geographical factor (latitude and UV availability), each country has its own dietary recommendation, vitamin D food fortification strategies and supplementation policy. Likewise, each subgroup or ethnic groups has a different eating habit, clothing, skin colour, physical activity, attitude towards sun exposure and genetic make-up. A combination and interaction of these factors may lead to a risk of VDD in the population. Therefore, there is a need to explore various factors that can increase the risk of VDD for a different country, ethnics, and sub-groups. In this study, the demographic characteristics, lifestyle factors and *GC* polymorphism that might associated with VDD were comprehensively assessed.

Despite abundance of sunlight throughout the year, no association between time spent outdoors and maternal VDD was noted in this study, likewise the skin type variable. These null results are consistent with that reported in previous local studies, which examined factors associated with VDD in early pregnancy [15]. No association was reported between Fitzpatrick classification, melanin indices, sun protection score and sunlight exposure with maternal VDD [15]. The null results could be due to a high proportion of Malay ethnic group in the study with majority of them (82.5%) were veiled. In support of this, veiled was identified as a risk of VDD in our study where veiled women were about 4 times at higher risk than unveiled women as UVB rays do not transmit through clothing. This finding is in an agreement with a study conducted in Saudi Arabia [50] where the researchers reported that veiled clothing was significantly associated with increased risk of VDD.

The positive association between vitamin D intake from food and supplements and maternal VDD is in agreement with most of the previous findings [40, 48, 51-56]except a study [46]. It was found that vitamin D intake from foods and supplements contributed significantly to 25OHD level in population where exposure to sun is minimum or dermal synthesis of 25OHD is limited. For instances, recent studies from two high latitude countries, Sweden [57] and Switzerland [40, 55]reported that consumption of supplements but not time spent outdoors was associated with decreased risk of VDD. Similarly, in a large Chinese study, Yun and colleagues [47] demonstrated that vitamin D supplement consumption was positively associated with vitamin D status during winter when the exposure to sunlight is limited but not significantly associated during autumn. In the current study, it was found that exposure to sun did not contribute to the risk of VDD, but dietary vitamin D intake did which is consistent with the findings by Yun and colleagues [47].

Previous studies that investigated the association of *GC* rs7041 with 25OHD showed that C allele was associated with decreased risk of VDD among pregnant [25-27, 58] and non-pregnant population [19–22]. In contrast to the previous studies, the C allele was found to increase the risk of VDD among pregnant women in the current study. The discrepancy could be due to the factors included in this study were different. For instance, the previous studies examined the factors associated with maternal VDD have been restricted to the only environmental [15, 23, 24] or genetic variables alone [25–28]. Additionally, it is possible that changes in the metabolism of vitamin D notably the elevation of circulating vitamin D binding protein concentration may change the association of *GC* SNP with vitamin D status. This could be explained by the drastic change in the metabolism of vitamin D during pregnancy that causes variation in the factors associated with VDD during pregnancy compared to the non-pregnant state.

This study has several limitations as the data was collected in a public hospital where the findings could not be generalised to the pregnant women in private hospital. However, this study assessed a wide range of possible factors associated with maternal VDD: vitamin D intake from diet and supplements, estimates of sun exposure, skin type, clothing and SNPs. Owing to the limited resources, the sample size of this study was small which limited the number of SNPs that could be studied in the present study. However, two SNPs (rs4588 and rs7041) that were mostly reported to be associated with 25OHD in the previous studies were selected and investigated in this study. The sample size used in this study had a sufficient power to detect the prevalence and the associations of the demographic characteristics, lifestyle and GC SNPs (or diplotypes) with maternal VDD. To date, there is lack of consensus on the cut-off values used to define VDD. It is important to note that the results of factors associated with VDD in the present study might different if a different cut-off was used. However, the cut-off used in the present study was based on careful review by National Academy of Medicine (formerly known as Institute of Medicine) that suggests 25OHD level < 30 nmol/L is at increased risk of rickets, impaired fractional calcium absorption and decreased bone mineral content.

The high prevalence of maternal VDD reported in this study indicates the need for urgent development and implementation of strategies to improve maternal vitamin D status. In Malaysia, where sunlight is available throughout the year, advocating increasing sunlight exposure will be a cost-effective measure. Nonetheless, as a large majority of Malaysian women are veiled, advocating sun exposure may not increase the 25OHD level in majority of them. Veiled is a non-modifiable risk factor as almost all of the Malay women are veiled as part of religious requirement. Given that the food source of vitamin D is limited, it is a potential strategy to increase 25OHD level in pregnant women by vitamin D supplementation where a significant positive association of vitamin status with vitamin D intake from the supplement was found in this study. However, less than half (40.1%) of the study participants consumed supplements containing vitamin D where prenatal multivitamin was the most prevalent supplement (66.7%) consumed. Most of the pregnant women obtained their prenatal multivitamins (containing 10 μg of vitamin D per capsule) from public health clinics during their antenatal visit while few of them bought the supplement themselves. It should be noted that in the Malaysian public health clinic, the provision of prenatal multivitamin is not universal. However, the provision is dependent on the availability and the requirement of a pregnant women. Universal provision of a prenatal multivitamin may be effective in improving the vitamin D status of pregnant women. Investigating the effectiveness of supplement consumption by pregnant women in randomised controlled trials could be the next step to develop national recommendations or policies for supplementation. Additionally, the significant association of *GC* SNPs (and *GC* diplotypes) with maternal VDD showed in the present and previous studies suggests that supplementation should be more personalised based on genotype and risk factors assessment, particularly in pregnant women.

## Conclusions

Almost half of the pregnant women participated in this study were VDD (25OHD < 30 nmol/L). Sun exposure was found not contribute to maternal vitamin D status. The contribution of *GC* rs4588 and rs7041 (or *GC* diplotypes) to maternal VDD was minimal but was significant. The findings showed that the risk of VDD was 4 times higher in veiled than unveiled women whereas vitamin D intake from foods and supplements were significantly associated with maternal VDD. Veiled clothing is a non-modifiable risk factors as majority of participants were veiled for religion requirement. Therefore, the current research suggests that food fortification and supplementation could be the potential strategy to improve vitamin D status among pregnant women.

## Supplementary Information

**Additional file 1.** Questionnaire used in this study.

## Data Availability

The datasets used and/or analysed during the current study are available from the corresponding author on reasonable request. The questionnaire used in this study (supplementary file 1) are uploaded as supplementary material.

## Abbreviations

25OHD: 25-hydroxyvitamin D
aOR: Adjusted odd ratio
BMI: Body Mass Index
BSA: Body surface area
CI: Confidence interval
CYP27B1: Cytochrome P450-27B1
CYP2R1: Cytochrome P450-2R1
DNA: Deoxyribonucleic acid
FFQ: Food Frequency Questionnaire
GC: Vitamin D binding protein
GWAS: Genome-wide association studies
HWE: Hardy- Weinberg Equilibrium
IOM: Institute of Medicine
IQR: Interquartile range
LMP: Last menstrual period
PCR: Polymerase Chain Reaction
RFLP: Restriction fragment length polymorphism
SD: Standard deviation
SNPs: Single nucleotide polymorphisms
UHPLC: Ultra-High-Performance Liquid Chromatography
VDD: Vitamin D deficiency
