# Supplementary material for: Influence of vitamin D binding protein polymorphism, demographics and lifestyle factors on vitamin D status of healthy Malaysian pregnant women
Source: BMC Pregnancy Childbirth. 2020 Nov 23;20:714. doi: 10.1186/s12884-020-03397-7 (PMC7684735; doi:10.1186/s12884-020-03397-7)
Supplement: Supplementary file 1 — Additional file 1. Questionnaire used in this study. [file 12884_2020_3397_MOESM1_ESM.pdf]

Medical records

Date of recruitment:

Subject ID:

**Personal details**				
1.	Age		LMP	
2.	Ethnicity		EDD	
3	Education level		Gravidity, Parity, Abortion (e.g. G1P0)	
4	Occupation		Partner's occupation	
5	Family H/O		Previous pregnancy	
6.	Height			
**Booking**		**First booking**	**Last booking**	
1	Date @ week			
2	Weight			
3	Hb			
4.	Ultrasound scan	CRL:	BPD:<br>HC:<br>AC:<br>FL:	
**Deliver**				
1.	Date time		Week	
1	Birth weight (kg)		APGA 1 min	
2	Length (cm)		APGA 5 min	
3	HC (cm)		MOD	
4	sex			

Date: __ __ / __ __ / __ __ __ __ (Day / Month / Year)

PART A : Socio demographic characteristic

**A1**	What is your **date of birth**? (dd/mm/yyyy)	☐☐ / ☐☐ / ☐☐☐☐
**A2**	**How old** are you?	☐☐ **years**
**A3**	What is your current **marital status**?	☐ 1. Single<br>☐ 2. Married<br>☐ 3. Divorced<br>☐ 4. Widowed<br>☐ 5. Cohabiting
**A4**	What is your **ethnic group background**?	☐ 1. Malay<br>☐ 2. Chinese<br>☐ 3. Indian<br>☐ 4. Others (Please state: ....)
**A5**	What is the **highest level of education** that you have completed?	☐ 1. No formal schooling<br>☐ 2. Primary school (1 /2 /3 /4 /5 /6)<br>☐ 3. Secondary school (1/2/3/4/5)<br>☐ 4. Pre-U (STPM, matriculation, diploma.)<br>☐ 5. University/ College (1/2/3/4/5)<br>☐ 6. Post graduate degree completed (Master/PhD)
**A6**	How **many years of school**, including higher education have **you completed**?	☐☐ **years**
**A7**	What is your **current job?**	☐ 1. Not working for pay → **Go to question A11**<br>☐ 2. Government employee<br>☐ 3. Non-government employee<br>☐ 4. Self-employed /Employer
**A8**	**What is your main occupation?**	

Date: Day | Month | Year

**A9**	**Where** do you spent most of your working time ?	☐ 1. Indoor<br>☐ 2. Outdoor<br>☐ 3. Both
**A10**	What is your **monthly income** ?	☐ RM0-1500<br>☐ RM1501-3000<br>☐ RM3001-5000<br>☐ RM5001-8000<br>☐ RM8001 and above
**A11**	What is the main **reason** you are not working for pay?<br>***(Skip if you are working)***	☐ 1. Homemaker/ caring for family<br>☐ 2. Looked but can't find a job<br>☐ 3. Doing unpaid work/ voluntary activities<br>☐ 4. Studies/training<br>☐ 5. Retired<br>☐ 6. Ill health<br>☐ 7. Others (Please state:..................................)
**A12**	What is **your husband/ partner's ethnic group background**?	☐ 1. Malay<br>☐ 2. Chinese<br>☐ 3. Indian<br>☐ 4. Others (Please state: ....)
**A13**	What is the **Highest level of education** that **your husband/ partner** have completed?	☐ 1. No formal schooling<br>☐ 2. Primary school (1 /2 /3 /4 /5 /6)<br>☐ 3. Secondary school (1/2/3/4/5)<br>☐ 4. Pre-U (STPM, matriculation, diploma.)<br>☐ 5. University/ College (1/2/3/4/5)<br>☐ 6. Post graduate degree completed (Master/PhD)

Date: | | | | | | | | |
Day Month Year

**A14**	What is your **husband/ partner current job?**	☐ 1. Not working for pay<br>☐ 2. Government employee<br>☐ 3. Non-government employee<br>☐ 4. Self-employed<br>☐ 5. Employer
**A15**	What is **your husband/partner <u>main occupation</u>?**	
**A16**	What is **your husband/partner <u>monthly income</u> ?**	☐ RM0-1500<br>☐ RM1501-3000<br>☐ RM3001-5000<br>☐ RM5001-8000<br>☐ RM8001 and above
**A17**	What is your **<u>total household income</u>** (including your income and the income of your husband/partner and income from other sources)?	☐ RM0-1500<br>☐ RM1501-3000<br>☐ RM3001-5000<br>☐ RM5001-8000<br>☐ RM8001 and above

PART B: Pregnancy history and details

**B01**	Regardless of the pregnancy outcome, **<u>how many times</u>** you have been pregnant?<br>*(include your current pregnancy)*	.... Times
**B02**	**<u>How many times</u>** you have given birth to a live baby?	.... Times
**B03**	Did you ever give birth to a baby that weighed **<u>less than 2.5kg</u>** (5Ibs, 8ozs)?	☐ 1. Yes<br>☐ 2. No

Date: |_|_|_|_|_|_|_|_| (Day / Month / Year)

**B04**	Did you ever have a baby that was born **prematurely** (before you completed 37 weeks of pregnancy)	☐ 1. Yes<br>☐ 2. No
**B05**	How many times you have **abortion**?<br>(*Include both induced and spontaneous abortion)*	.... Times
**B06**	At what **age** did you **get your first pregnancy**?	.... Years old
**B07**	Just **before you got pregnant**, how much did you **weigh**?	.... Kilograms
**B08**	About how much do you **weigh** **right now**?	.... Kilograms
**B09**	How **tall** are you without shoes?	.... cm
**B10**	**Before you got pregnant**, did a doctor, nurse or other health care worker ever tell you that you had any of the following **health condition**?	☐ No<br>☐ Diabetes (High blood glucose)<br>☐ Hypertension (High blood pressure)<br>☐ Cardiovascular disease<br>☐ Renal disease<br>☐ Others (Please state: ....)...
**B11**	During your **current pregnancy**, did a doctor, nurse or other health care worker ever tell you that you had any of the following **health conditions**?	☐ No<br>☐ Gestational Diabetes<br>☐ Pre-eclampsia<br>☐ Eclampsia<br>☐ Anemia<br>☐ Endometriosis<br>☐ Placenta Abruption<br>☐ Others (Please state: ....).

Date: | Day | Month | Year |

PART C: Smoking

**C01**	Are you a smoker?	☐ 1. No. **(skip PART C)**<br>☐ 2. Yes, I was a former smoker.<br>☐ 3. Yes, i am currently smoke.
**C02**	Have you smoked any cigarettes in the past 2 years?	☐ 1. Yes<br>☐ 2. No
**C03**	During the 3 months before you got pregnant, how many cigarettes did you smoke on an average day?	.... sticks
**C04**	During the first 3 months of your pregnancy (including before you knew you were pregnant), how many cigarettes did you smoke on an average day?	.... sticks
**C05**	How many cigarettes do you smoke on an average day now?	.... sticks

PART D: Skin Typing

Please fill out the questionnaire similar to this example:

- *If the colour of your eyes are dark brown, then mark the answer as shown below.*

**Score**	**0**	**1**	**2**	**3**	**4**
What are the colour of your eyes?	Light blue, gray, green	Blue, gray or green	Blue	Dark brown (marked)	Brownish black

D01. Genetic Disposition

**Score**		**0**	**1**	**2**	**3**	**4**
**a.**	What are the colour of your eyes?	Light blue, gray, green	Blue, gray or green	Blue	Dark brown	Brownish black
**b.**	What is the natural colour of your hair?	Sandy red	Blond	Chestnut/ Dark, blond	Dark brown	Black
**c.**	What is the color of your skin (non exposed areas)?	Reddish	Very pale	Pale with beige tint	Light brown	Dark brown
**d.**	Did you have freckles on unexposed areas?	Many	Several	Few	Incidental	None

Date: | Day | Month | Year |

D02. Reaction to Sun Exposure

	Score	0	1	2	3	4
a.	What happens when you stay in the sun too long?	Painful redness, blistering, peeling	Blistering followed by peeling	Burns sometimes followed by peeling	Rare burns	Never had burns
b.	To what degree do you turn brown?	Hardly or not at all	Light colour tan	Reasonable tan	Tan very easy	Turn dark brown quickly
c.	Do you turn brown within several hours after sun exposure?	Never	Seldom	Sometimes	Often	Always
d.	How does your face react to the sun?	Very sensitive	Sensitive	Normal	Very resistant	Never had a problem

D03. Tanning Habits

	Score	0	1	2	3	4
a.	When did you last expose your body to sun (or artificial sunlamp/ tanning cream)?	More than 3 months ago	2-3 months ago	1-2 months ago	Less than a month ago	Less than 2 weeks ago
b.	Did you expose the area to be treated to the sun?	Never	Hardly ever	Sometimes	Often	Always

Date: Day | Month | Year

PART E: Sun Exposure Log

Instructions:

1) Record how long you were outdoors during each time period if it is **greater than 5 mins.**
   - For example: If you were outside from 1:33pm to 1:55pm, note 22 mins. in the time period from 1pm-2pm.
2) Record your outdoor activity next to the appropriate time period.
   -For example: walk, bike, run, sit, stand, swim, garden
3) Record which parts of your body were exposed to the sun or covered with sunscreen using the key provided. (Please refer to the attached clothing key)
4) Record your sun exposure frequency (per week) and fill out the form similar to this **example**:

Time of day	Outdoor activity	Time spent outdoors (minutes)	Frequency	Are you using?		Clothing				Sunscreen				SPF
				Glove	Umbrella	A	B	C	D	A	B	C	D	
7am-8am	Jogging	40	5/7			1	3	4	3	✓				15
8am-9am	Walking	10	7/7		✓	1	4	3	2					

Time of day	Outdoor activity	Time spent outdoors (minutes)	Frequency	Are you using?		Clothing				Sunscreen				SPF
				Glove	Umbrella	A	B	C	D	A	B	C	D	
7am-8am														
8am-9am														
9am-10am														
10am-11am														
11am-12pm														
12pm-1pm														
1pm-2pm														
2pm-3pm														
3pm-4pm														
4pm-5pm														
5pm-6pm														
6pm-7pm														

PART F: Food frequency questionnaire

	Food item		Name of brand/product	Frequency of intake				Serving size	Total serving every meal
				How many times a day	How many times a week	How many times a month	Never @ < once a month		
**A.**	**Fortified food**								
1.	Milk powder	Full cream						Tablespoon<br>Scoop	
		Low fat						Tablespoon<br>Scoop	
		Skim						Tablespoon<br>Scoop	
2.	Prenatal milk							Tablespoon<br>Scoop	
3.	Fresh milk	Full cream						Box S/L<br>Cup/ Glass	
		Rendah lemak						Box S/L<br>Cup/ Glass	
		Low fat						Box S/L<br>Cup/ Glass	
4.	UHT milk							Box<br>S/L	
5.	Sterilized milk							Cup/ Glass	
6.	Sweetened condensed milk *(in tea, coffee or other beverages)*							Tablespoon/ teaspoon	
7.	Evaporated milk *(in tea, coffee or other beverages)*							Tablespoon/ teaspoon	
8.	Malt milk powder							Tablespoon	
9.	Milk with cereal (3 in 1)							Tablespoon/ pack	
10.	Cereal without milk							Tablespoon	
11	Soy drink							Box S/L<br>Cup/ Glass	
12	Glucose drink fortified with vitamin D							Tablespoon	
13	Yogurt (milk or soy)							Cup<br>Container	
14	Cultured milk							Bottle	

	Food item		Name of brand/product	Frequency of intake				Serving size	Total serving every meal
				How many times a day	How many times a week	How many times a month	Never @ < once a month		
15	Cheese							Piece<br>cup	
16<br>.	Biscuit							Piece<br>Packet	
17	Margarine (in any food eaten)							Teaspoon/<br>Tablespoon	
18	Butter (in any food eaten)							Teaspoon/<br>Tablespoon	
19	Bread							Piece	
20	Homemade ice cream made from milk							Cup<br>scoop	
21	Milk dessert (pudding @ custard)							Cup<br>scoop	
22	Waffle							Piece	
23	Pancake							Piece	
24	Potatoes; mashed with milk and margarine							Cup	
25	Lasagna, spaghetti with cheese							Cup	
26	Cream soup/ mushroom soup	Canned						Cup	
		Instant						Pack	

	Type of food and drink	Frequency of intake				Serving size	Total serving every meal
		How many times a day	How many times a week	How many times a month	Never @ < once a month		
B	**Natural Food (Seafood/ meat/ egg product/ mushroom)**						
1.	Canned mackerel					*piece*	
2.	Canned sardines					*piece*	
3	Fresh sardines						
4.	Canned Tuna					*piece*	
5.	Salmon					*piece*	
6.	Ikan kembong, Mackerel					*piece*	
7.	Ikan keli/ catfish					*piece/* slice	
8.	Tamban bulat, *Herring, round*					*piece/* slice	
9.	Ikan Cencaru,/ *Scad hardtail*					*piece/* slice	
10	Ikan selar bulat/ *Big ege scad*					*piece/* slice	
11	Ikan selar kuning/ *scad yellowtail*					*piece/* slice	
12.	Ikan selar/ *spotted travelley*					*piece/* slice	
13.	Ikan ekor kuning/ *Yellowtail*					*piece/* slice	
14.	Kerapu merah/ red grouper					*piece/* slice	
15.	Ikan bawal putih/ white promfret					*piece/* slice	
16.	Ikan bawal hitam/ black promfret					*piece/* slice	
17.	Ikan belanak/ Mullet bluetail					*piece/* slice	
18.	Ikan puyu/ Perch, climbing					*piece/* slice	
19.	Ikan tenggiri papan/ Mackerel spanish					*piece/* slice	
20.	Ikan tongkol					*piece/* slice	
21.	Ikan Lee Koh/ Carp common					*piece/* slice	
22.	Ikan parang/ Herring wolf					*piece/* slice	
23	Ikan kerapu, Grouper					*piece/* slice	
24	Cod					*piece/* slice	
25	Ikan jenahak/ white snapper					*piece/* slice	
26	Ikan siakap/ Perch gain sea					*piece/* slice	
27	Other fish: if any					*piece/* slice	
28	Ikan bilis/ anchovies					Cup	
29	Udang/ *prawn*					Piece	
30	Udang kecil/ shrimp					Tablespoon	
31	Kerang/ clam					Tablespoon	
32	Seafood others						

	Type of food and drink			Frequency of intake				Serving size	Total serving every meal
				How many times a day	How many times a week	How many times a month	Never @ < once a month		
B	Natural Food (Seafood/ meat/ egg product/ mushroom)								
33	Chicken	With skin						Piece	
		Without skin						Piece	
34	Beef	With skin						Box	
		Without skin						Box	
35	Pork/ Mutton	With skin						Box	
		Without skin						Box	
36	Beef sausage/ frankfurter							Piece	
37	Pork/ Chicken sausage/ frankfurter							Piece	
38	Ham/ luncheon meat							Piece	
39	Internal organ	Liver						Piece	
		Kidney						Piece	
		Lung						Piece	
40	Eggs: eaten alone or in foods		Egg white only					Piece	
			Egg yolk only					Piece	
			Whole					Piece	
41	Mushroom		Fresh					Cup	
			Dried					Cup	
			Canned					Cup	

D3	Makanan tambahan/ *Dietary supplement*	Kekerapan pengambilan/ *Frequency of intake*				Saiz sajian */serving size*	Jumlah sajian Setiap kali makan/ *Total serving every meal*
		**Berapa kali sehari** *How many times a day*	**Berapa kali seminggu** *How many times a week*	**Berapa kali sebulan** *How many times a month*	**Tidak pernah @ kurang dari sekali sebulan/** *Never @ < once a month*		
a	Minyak hati ikan cod<br>Cod liver oil<br>Jenama/*brand*:____<br>Brp lama/*how long*:___					Biji/*pill*<br>s.makan/. *t.spoon*	
b	Minyak ikan / Fish oil<br>Jenama/*brand*:_______<br>Brp lama/*how long*:___					Biji/*pill*<br>s.makan/. *t.spoon*	
c	New Obimin<br>Brp lama/*how long*:___					Biji/*pill*	
d	Obimin Plus<br>Brp lama/*how long*:___					Biji/*pill*	
e	Pramilet<br>Brp lama/*how long*:___					Biji/*pill*	
f	Iberet folic<br>Brp lama/*how long*:___					Biji/*pill*	
g	Vitamin D<br>Jenama/*brand:*_______<br>Brp lama/*how long*:___					Biji/*pill*	
h	Multivitamin<br>Jenama/*brand*:_______<br>Brp lama/*how long*:___					Biji/*pill*<br>s.makan/. *t.spoon*	
i	Kalsium dengan Vitamin D/ *Calcium with vitamin D supplements*<br>Jenama/*brand:*_______<br>Brp lama/*how long*:___					Biji/*pill*	
j	Kalsium tambahan<br>*Calcium supplements*<br>Jenama/*brand:*_______<br>Brp lama/*how long*:___					Biji/*pill*	
k	Vitamin C					Biji/*pill*	
l	Vitamin B kompleks					Biji/*pill*	
m	Asid folik/ Folic acid					Biji/*pill*	
n	Lain-lain/*Others*<br>*Jenama/brand:*_______					Biji/*pill*<br>s.makan/.	

* Senarai vitamin-vitamin yang sering diambil ketika hamil.
